# Evaluation of the CPR video decision aid with patients with end stage renal disease

**DOI:** 10.1186/s12882-018-1018-y

**Published:** 2018-09-12

**Authors:** Cherie Kapell Brown, Jennifer Kryworuchko, Wanda Martin

**Affiliations:** 1 Manager 5A Surgery and Ambulatory Care, St. Paul’s Hospital, 1702 20th Street West, Saskatoon, SK S7M OZ9 Canada; 2 School of Nursing and Centre for Health Services and Policy Research, The University of British Columbia, T275- 2211 Wesbrook Mall, Vancouver, BC V6T 2B5 Canada; 3 College of Nursing, University of Saskatchewan, PO Box 6, 104 Clinic Place, Saskatoon, SK S7N 2Z4 Canada

**Keywords:** Decision-aid, Quality of communication, CPR, End-of-life, Shared decision-making

## Abstract

**Background:**

People with end stage renal disease (ESRD) face important health-related decisions concerning end-of-life care and the use of life-support technologies. While people often want to be involved in making decisions about their health, there are many challenges. People with advanced illness may have limited or wavering ability to participate fully in decision-making conversations – or lack decisional capacity for making decisions. Additionally, they may have a limited understanding of CPR and tend to receive inconsistent information on the process and outcome of CPR. Unfortunately, these discussions are often avoided. Shared decision-making approaches are an approach to overcoming these challenges. The objectives of this research was to design, test, and analyze a novel CPR video decision aid (VDA) with nephrology patients and their families in a clinical setting.

**Methods:**

The Interprofessional Shared Decision-making Model was used as a framework to guide the research. A prospective quasi-experimental design included pre/posttest measures of knowledge and confidence in decision-making, and posttest only measure of uncertainty about the decision.

**Results:**

Participant knowledge about CPR increased from a mean score of 4.8/9 (standard deviation [SD] = 1.65) before viewing the video to 7.5/9 (SD = 1.40) (*p* = 0.000) after viewing the video. Decisional self-efficacy improved slightly from 84% pre intervention (SD 17.04, range 20–100) to 86% after the intervention (SD 14.13, range 39–100) (*p* = 0.005) for patient participants. Before the intervention, most patients (43/49; 86%) had an order to have CPR in the physician orders and very few (7/49; 14%) had an order not to have CPR. Immediately after viewing the CPR-VDA and completing the values clarification worksheet, fewer 28/49 (57%) chose to have CPR, 13 (27%) chose not to have CPR and 8 (16%) were unsure.

**Conclusions:**

The CPR-VDA was feasible and acceptable to patients with ESRD, their families and the healthcare team. The CPR-VDA positively affected decision-making: improving patient and family knowledge about CPR, clarity of values, patients’ decisional self-efficacy, the congruence between documented physician’s orders and patient choice, quality of communication about CPR, while reducing decisional conflict (uncertainty) amongst patients, families, and physicians.

## Background

Patients with end stage renal disease (ESRD) face important health related decisions, such as decisions about treatment options, palliative end-of-life care and whether life-support technologies are wanted in their care [1]. ESRD is the final stage of kidney disease where kidneys are working at less than 15% of their normal capacity, therefore requiring intervention to sustain life [2]. All patients with ESRD have had to make decisions through the course of their chronic illness, for example, about screening and diagnostic tests, vascular access, or dialysis modalities. The use of an additional life supportive technology such as cardiopulmonary resuscitation (CPR), if cardiac or respiratory arrest occurs is another important decision for people with ESRD during their healthcare journey. The aim of this study was to improve the quality of communication during CPR decision-making processes involving patients with ESRD, their families and the healthcare team.

Patients with ESRD have a higher incidence of cardiac arrest because dialysis treatment exacerbates cardiac disease. Healthcare providers may offer CPR to try to restore cardiac and respiratory function and prolong life [3]. Unfortunately, CPR does not work very well for patients with advanced medical illness like ESRD and has poor prognosis [4, 5]. Higher initial survival can be observed after cardiac arrest in a hemodialysis unit compared to other hospital settings and this may be attributed to availability of equipment, personnel, and vascular access [5]. However, overall survival after cardiac arrest for older adults, regardless of setting, with advanced chronic illness such as kidney disease remains poor, with less than 20% of patients who receive CPR surviving to hospital discharge [6–8].

Jones, Podolsky, and Green [9] argue that the health care system has not responded with sufficient education, engagement, and support in the decision-making process. As patients and family members increasingly are called upon to make complex decisions, they are challenged by a lack of medical knowledge, low health literacy, and uncertainty about their prognosis. Indeed, for medical, social, cultural, and legal reasons, many physicians are reluctant to meaningfully engage patients nearing end-of-life in advance care planning [9]. What has followed is a potentially unwanted increase in technological care at the end-of-life.

For example, when asked, patients maintain they would prefer less aggressive care if death were likely in the short term [10]. ESRD patients and their families feel they are not always part of discussions around prognosis, treatment goals, and end-of-life care [11, 12]. Almost 30% of patients over 75 years of age prefer to stop dialysis because they experience unacceptably poor quality of life and therapy intolerance [13]. There is also increasing awareness that resources and spending at end-of-life are not well correlated with quality of life or quality of care [14]. Canadians, older patients, and health care professionals increasingly are focused on meaningful advance care planning and promoting a shared, informed decision-making process about the use of technology at the end-of-life.

The CPR video decision aid (VDA) is a novel approach to shared decision-making preparing patients, families, and their healthcare professionals for conversations about the CPR decision. Patient decision aids are resources that can identify evidence-based healthcare options, their benefits and harms, and further assist patients to communicate their values and preferences to their healthcare provider [15]. With the use of decision aids, patients have a better understanding about care options and anticipated outcomes, have improve perceived risk, experience a good match between values and choices, and show a reduction in indecision and regret that leads to decisional conflict [16].

This study consisted of field-testing the CPR-VDA in a community context with ESRD patients who have already made a similar complex decision to receive dialysis. The study was part of a larger research program aimed at improving the quality of communication during decision-making processes involving patients with advanced illness, their families, and the healthcare team. The aim of this study was twofold: to improve patient and family member knowledge and involvement in decisions about CPR, and to ensure that CPR was provided only when wanted. The hypothesis was that the CPR-VDA would be acceptable to patients and feasible to use to prepare ESRD patients for shared decision-making about CPR. The video would also improve patient and family knowledge, clarify values, and thus improve congruence between documented orders and patient choice. The patient and their family member would experience minimal residual uncertainty about the decision thereby improving confidence to make the decision.

## Methods

### Design

A prospective quasi-experimental design included pre/posttest measures of knowledge and confidence in decision-making, and posttest only measure of uncertainty about the decision (decisional conflict). Research Ethics Board approval was obtained prior to commencing the study (BEH 13–200) and all participants provided written informed consent. The study was also approved by the local patient and family advisory council and health authority partners.

### Setting and participants

Participants were recruited between late summer and mid-winter 2015 in an urban inpatient and outpatient hemodialysis center. At these centers, 260 patients receive hemodialysis 6 days per week. A convenience sample of eight physicians who cared for patients with ESRD receiving dialysis was recruited from the hemodialysis centers. Eligible physicians were staff physicians (nephrologists) or residents on their nephrology rotation. Recruited physicians and members from the renal health care team (social worker, clinical nurse specialist, and unit manager) helped identify eligible patients in the hemodialysis program and/or family members and introduced the study. The research nurse then determined the final eligibility of patients and family members who expressed desire to participate.

Eligible patients were over 55 years of age, had stage 5 renal failure, were dependent on dialysis, and could communicate in English. Patients were invited to identify the adult ‘family’ member who knew them best, including partners, significant others, and/or close friends. The family member had to be 18 years or older, speak and understand English, have capacity to make healthcare decisions, have accompanied the patient to dialysis at least once, and have provided assistance to the patient without pay. Ideally, we wanted to recruit dyads consisting of a patient and their family member with whom they shared healthcare decisions. However, willing patients who could consent were included even without the participation of a family member. Patients without decisional capacity were invited to assent to participate if their family member was a participant.

### Intervention

The CPR-VDA is a seven-minute video designed for participants to independently view on a portable screen. The video presents information about CPR and the alternative option (comfort care) as well as information about the patient experience and important health outcomes. A CPR decision worksheet, which included a values clarification exercise, tailored the generic patient decision aid format to the CPR decision, and was completed with the study nurse. A plain language script for the CPR-VDA was a significant adaptation from an earlier paper-based CPR information tool [17], and was informed by a rapid systematic review process of CPR outcomes data. In collaboration with the research team, a cinematographer produced the final video CPR-VDA that is publicly available at http://vimeo.com/48147363. The study nurse provided non-directive support to help participants complete the questionnaires thus preparing them for the discussion with the physician, consistent with the role of a decision coach in a shared decision framework [18].

### Procedure

The study nurse conducted interviews during a scheduled dialysis treatment. The interview commenced with the patient and/or family member once dialysis was initiated and the patient remained physically stable (Table 1). After obtaining consent, a brief chart review was conducted to check documented CPR orders and pertinent health history. Interviews occurred in a semi private place with patients, family, and staff in close proximity.

**Table 1:** Data Collection Strategy

Data source	Time period for data collection	Collection tool
Participating Patient / Family	Pre VDA intervention	PART A: Demographics, Frailty Index, Health Literacy Score Knowledge about CPR Decisional Self-efficacy
	CPR-VDA Intervention	View CPR-VDA (http://vimeo.com/48147363) Observation Matrix (i.e., elements of capacity, fatigue, attention)
	Post VDA intervention	PART B: Acceptability Survey Knowledge about CPR Decisional Conflict Scale Decisional Self-efficacy Scale CPR Worksheet
Study Nurse	Patient / Family / Physician participants discuss CPR decision	OPTION
Participating Physician	Post VDA intervention and discussion	PART A: Demographics (completed once only per physician) PART B: Physician Survey (completed after engaging in each discussion about CPR: Decisional Conflict Scale)
Medical record of participating patient	At enrollment (consent) and 1 week from date of enrollment	Chart Abstraction Tool (co-morbid illnesses, the presence of ‘Goals of Care’ orders, ‘DNR’ orders, ‘Resuscitation Care-plan’ orders and any order related to CPR)
Study Nurse	Initiated at time of enrollment until end of participation	Field notes

The first part of the questionnaires included; demographics with a self-reported frailty index and health literacy test, pre-knowledge test about CPR and pre-intervention self-efficacy questions. Following this series of questions, the patient and/or family member viewed the seven-minute CPR-VDA video on an iPad screen. The interview continued with acceptability questions regarding the CPR-VDA video, post-knowledge test questions about CPR, post-decision self-efficacy questions and series of questions to assess any decision conflict related to the CPR decision. The study nurse asked patients their CPR preference after viewing the CPR-VDA and completing the values clarification worksheet.

### Outcomes

Acceptability was assessed using eight validated questions about use, amount of information, the length, the clarity, balance in presentation, willingness to recommend to others and overall suitability for decision-making [19, 20]. Knowledge about CPR was tested using nine questions developed by the research team. Self-Efficacy is the participants’ self-confidence or belief in their abilities in decision-making, and was measured using the Decision Self-Efficacy Scale [21]. The scale gave a total score out of 100, and a higher score indicates higher self-efficacy for decision-making. Used to evaluate a decision aid for women with osteoporosis, the reliability of the scale was 0.92 and it correlated with decisional conflict subscales of feeling informed (*r* = 0.47) and supported (*r* = 0.45) [21]. Decision conflict was also assessed, which occurs when a patient is uncertain about what course of action is best for them [22]. The perception of uncertainty is related to modifiable factors such as feeling uninformed, being unclear about personal values for the options, or feeling unsupported in decision-making [22]. Decision conflict was measured using the low literacy version of the Decision Conflict Scale for patient/family [22] where scores lower than 25/100 were associated with decision implementation and scores over 37.5/100 were associated with delayed decisions. The scale has a reliability of 0.78 and has been used in many studies of patient decision aids [16, 22]. Decisional conflict was also measured using the SURE test [23] in the values clarification worksheet. The reliability of this scale is 0.65 [23]. Measurement of relevancy of the CPR decision for the patient at this stage of their healthcare journey was asked before and after viewing the video. After participant(s) completed the pre and posttest questionnaires, they were then invited to complete the paper based values clarification worksheet.

Once all patient/family questionnaires were completed, the physician caring for the patient discussed CPR with the patient and/or family member to assess whether or not they wanted CPR in the event of a cardiac or respiratory arrest. The research nurse observed this discussion and completed an Observing Patient Involvement (OPTION) tool that assessed physician and patient involvement in shared decision-making. The OPTION instrument was developed to evaluate shared decision-making communication and the reliability of this scale was 0.66 in a study evaluating physician encounters in primary care settings [24]. Responses on a five-point scale ranged from ‘the behavior is not observed’ to ‘observed and executed to a high standard’. The total summed score range from zero to 48 with higher scores indicating greater competency in shared decision-making [24]. Physicians completed a final questionnaire to report on the communication with patient/family. During the interview time, the study nurse used an observation matrix and field notes. The matrix captured observations such as time of day, distractions, and local environment, before and after the CPR-VDA intervention. The data collection strategy is summarized in Table 1.

### Statistical analysis

Baseline characteristics and outcomes were reported using proportions for categorical variables, and means and standard deviations (SD) for continuous variables. For each outcome of interest, analysis was conducted at the participant level using descriptive statistics and when appropriate, a paired sample t-test for comparing means (before/after). Data management and statistical analyses were conduct using IBM SPSS Statistics (Version 23).

## Results

### Demographics

Of those invited to participate, 8/8 (100%) physicians accepted, 49/53 (92%) patients accepted, and 8/9 (89%) family members accepted (Table 2). Of the five people who declined to participate, four were not interested and one patient was unable to complete the interview due to declining health status. There were seven patient/family member dyads and one family member who participated without the patient. Physicians were mostly experienced clinical nephrology staff and some had either palliative care experience or training about goals of care communication. Fewer than half of patients were female, half were married, and the average age was 67 years. Most patients lived in their own home in an urban setting where they received dialysis. Still, one third of the patients were from a rural area and travelled into the city for treatment. Patients had relatively high health literacy score despite having high school or less education. Over half of patients considered themselves vulnerable to severely frail and most had prior communication about CPR, which varied in formality.

**Table 2:** Demographics - Patient and Family

Demographic	Patient *n* = 49	Family *n* = 8
Age M (range, SD)	67 (55–91, 9.66)	62 (48–72, 8.19)
Female n (%)	21 (43%)	5 (63%)
Marital Status		
Married or living as married	28 (57%)	8 (100%)
Widowed	10 (21%)	0
Never married	5 (10%)	0
Divorced or separated	6 (12%)	0
Rural	15 (31%)	5 (63%)
Urban	34 (69%)	3 (37%)
Living Arrangement		
Home	36 (74%)	8 (100%)
Retirement residence	8 (16%)	0
Long-term care or nursing home	4 (8%)	0
Assisted living	1 (2%)	0
Highest Education		
Elementary school or less	3 (6%)	0
Some high school	17 (35%)	1 (12.5%)
High school graduate	11 (24%)	4 (50%)
Some college/trade school	8 (16%)	0
College/trade school diploma	2 (4%)	0
Some university	4 (8%)	1 (12.5%)
University graduate	4 (8%)	1 (12.5%)
Graduate degree	0	1 (12.5%)
Health Literacy (out of 8) M (range, SD)	6.61 (0–8, 2.42)	8 (8, 0)
Importance of Religion n (%)		
Extremely important	6 (12%)	1 (12.5%)
Very important	16 (33%)	3 (37.5%)
Somewhat important	14 (29%)	3 (37.5%)
Not very important	8 (16%)	1 (12.5%)
Not at all important	4 (8%)	0
Don’t know	1 (2%)	0
Prior communication about CPR? Yes	30 (61%)	6 (75%)
Patient Frailty		
Very fit	0	
Well	3 (6%)	
Managing well	19 (39%)	
Vulnerable	18 (36%)	
Mildly frail	5 (11%)	
Moderately frail	2 (4%)	
Severely frail	2 (4%)	

*M* mean, *SD* standard deviation

### Feasibility and acceptability

All patient and family member participants viewed the CPR-VDA and completed the values clarification worksheet during the interview time. Each had a follow-up discussion with their physician, although the discussion did not always occur on the same day as the initial interview. After viewing the CPR-VDA and completing the worksheet, participants were clear about the necessary decision, knew the options, could articulate their values, and could discuss these in varying degrees of detail. They were also able to identify their support person(s), decision-making needs and make a plan for next steps. During the video viewing, challenges included poor lighting, disruptive noise, physical discomfort (i.e., vascular access in their arm / needle in arm) positioning for treatment, fatigue, thirst, and confusion over wording. Multiple interruptions in the busy environment affected some people’s ability to attend to detail during the interview / intervention process resulting in 6% (3/49) of patients needing to review parts of the video. While working through the values clarification worksheet, participants revealed emotional struggles surrounding their current health state and concerns about the future.

Participants were asked how relevant the decision about CPR was for them before and after the intervention, and while the average rating was that it was relevant to them (2/5), the mean score increased from 2.1 to 2.3 (*p* = 0.01) after the intervention. Ninety-eight percent (56/57) of patient and family member participants rated the CPR-VDA as good to excellent. Seventy-seven percent (44/57) stated it contained the right amount of information, 75% (43/57) thought the information in the video was completely balanced, and 93% (53/57) found the information presented about CPR to be clear. The CPR-VDA was helpful in making decisions about CPR for 89% (51/57) of participants and almost everyone (98%) would recommend the video to other people who are considering CPR (Table 3).

**Table 3:** Acceptability of CPR-VDA

	Patient *n* = 49		
	Pre	Post	*p*-value
Relevance of the CPR decision M (range, SD) (Not relevant 0–1–2-3-4 Very relevant)	2.1 (0–4, 1.1)	2.3 (0–4, 1.1)	0.01
Item	Patient *n* = 49 n (%)		Family *n* = 8 n (%)
“How would you rate the CPR video decision aid?”			
Poor	0		0
Fair	1 (2%)		0
Good	19 (38%)		3 (37.5%)
Very good	20 (40.8%)		4 (50%)
Excellent	9 (18.4%)		1 (12.5%)
“How would you rate the amount of information in the video?”			
Much less than I needed	0		0
A little less than I needed	4 (8.2%)		0
About the right amount	37 (75.5%)		7 (87.5%)
A little more than I needed	6 (12.2%)		0
A lot more than I needed	2 (4.1%)		1 (12.5%)
“How balanced was the video’s information about CPR?”			
Clearly slanted towards having CPR	4 (8.2%)		0
A little slanted towards having CPR	4 (8.2%)		1 (12.5%)
Completely balanced	38 (77.6%)		5 (62.5%)
A little slanted towards not having CPR	3 (6.1%)		2 (25%)
Clearly slanted towards not having CPR	0		0
“How clear was the information in the video?”			
Everything was clear	29 (59.2%)		7 (87.5%)
Most things were clear	17 (34.7%)		0
Some things were clear	2 (4.1%)		1 (12.5%)
Many things were unclear	1 (2%)		0
“How helpful was the video in helping you make decisions about CPR?”			
Very helpful	28 (57.1%)		4 (50%)
Somewhat helpful	16 (32.7%)		3 (37.5%)
A little helpful	5 (10.2%)		0
Not helpful	0		1 (12.5%)
“Would you recommend this video to other people who are considering CPR?”			
I would definitely recommend it	40 (81.6%)		6 (75%)
I would probably recommend it	8 (16.3%)		2 (25%)
I would probably not recommend it	1 (2%)		0
I would definitely not recommend	0		0

### Effectiveness of the decision aid

Participant knowledge about CPR increased from a mean score of 4.8/9 (standard deviation [SD] = 1.65) before viewing the video to 7.5/9 (SD = 1.40) (*p* = 0.000) after viewing the video. Decisional self-efficacy improved slightly from 84% pre intervention (SD 17.04, range 20–100) to 86% after the intervention (SD 14.13, range 39–100) (*p* = 0.005) for patient participants; however, family members’ scores remained high in both periods (Table 4). Decisional conflict scores were relatively low overall (scores could range from 0 [no decisional conflict] to 100 [extremely high decisional conflict]); they were higher amongst patients (mean score of 13.57, SD = 18.34, range 0–70) but very low amongst family members (mean score of 1.25, SD = 3.54, range 0–10). Decisional conflict was also measured for patients using the clinical SURE test on the values clarification worksheet: 14 (28%) patients reported experiencing decisional conflict while 36 (72%) reported no decisional conflict.

**Table 4:** Effectiveness of the Decision Aid

Outcome	Patient *n* = 49			Family *n* = 8		
	Pre	Post	*p*-value	Pre	Post	*p*-value
CPR test questions n (%) correct answers						
1. When the heart stops beating, brain death will occur in: *several minutes.*	23 (47%)	40 (81%)		5 (63%)	6 (75%)	
2. CPR includes the following treatments: *pressing hard and fast on the breastbone to pump blood through the heart to the body.*	39 (80%)	46 (94%)		7 (88%)	8 (100%)	
3. If CPR is successful and the heart restarts the person: *usually needs a machine to help with breathing, medicines, and fluids while trying to recover in ICU (Intensive Care Unit).*	18 (37%)	39 (80%)		1 (13%)	6 (75%)	
4. The most serious possible harm from the heart stopping and needing to have CPR is: *severe brain damage from lack of oxygen*	32 (65%)	42 (86%)		4 (50%)	8 (100%)	
5. When CPR is effective it will: *restart the heart but have absolutely no effect on other medical conditions.*	25 (51%)	39 (80%)		8 (100%)	8 (100%)	
6. If 100 people have a chronic condition (heart failure, kidney failure, chronic lung disease) and their heart stops, how many will survive CPR and recover well enough to leave the hospital?: *very few people (10 out of 100).*	15 (31%)	43 (88%)		3 (38%)	8 (100%)	
7. If the patient decides NOT to have CPR: *they can receive treatments to relieve suffering AND for other medical conditions if wanted.*	25 (51%)	43 (88%)		6 (75%)	7 (88%)	
8. The healthcare team wants to talk to hospitalized patients about the CPR decision because: *the right decision about CPR depends on what is most important to the individual patient in addition to the patient’s medical conditions.*	28 (57%)	43 (88%)		5 (63%)	7 (88%)	
9. Of all the people who survive CPR, how many will have severe brain damage?: *a few survivors.*	30 (61%)	34 (69%)		5 (63%)	7 (88%)	
Knowledge (out of 9) M (range, SD)	4.8 (0–8, 1.65)	7.5 (4–9, 1.40)	0.000	5.6 (4–7, 1.31)	8.1 (6–9, 0.99)	0.000
Decisional Self-Efficacy (0 = extremely low; 100 = extremely high) M (range, SD)	84 (20–100, 17.04)	86 (39–100, 14.13)	0.005	86 (52–100, 15.98)	92 (77–100, 8.23)	0.203
Certainty						
Decisional conflict scale (0 = no conflict; 100 = high conflict) M(range, SD)		13.57 (0–70, 18.34)			1.25 (0–10, 3.54)	
SURE n (%)						
4 (no decisional conflict)		36 (72%)				
3		6 (12%)				
2		3 (6%)				
1		3 (6%)				
0 (high decisional conflict)		2 (4%)				
Preference n (%)						
Have CPR		28 (57%)				
No CPR		13 (27%)				
Unsure		8 (16%)				
Physician Order n (%)						
Have CPR = 1	43 (86%)	36 (72%)				
No CPR = 2	7 (14%)	14 (28%)				
M (range, SD)	1.14 (1–2, 0.35)	1.28 (1–2, 0.45)	0.007			
Observation of 50 single interactions between each patient/family and physician						
OPTION (score out of 48) M (range, SD)	25.66 (9–47, 7.41)					
Physician Exit Survey						
Relevance of the CPR decision for my patient M (range, SD) (Not relevant 0-1-2-3-4 Very relevant)	3.60 (2–4, 0.53)					
Satisfaction felt with discussion about CPR with patient M (range, SD) (Not at all 0-1-2-3-4 Completely)	3.18 (1–4, 0.79)					
Overall experience with the CPR discussion M (range, SD) (Very easy 0-1-2-3-4 Very Difficult)	0.80 (0–3, 0.80)					

Before the intervention, most patients (43/49; 86%) had an order to have CPR in the physician orders and very few (7/49; 14%) had an order not to have CPR. Immediately after viewing the CPR-VDA and completing the values clarification worksheet, fewer 28/49 (57%) chose to have CPR, 13 (27%) chose not to have CPR and 8 (16%) were unsure. Final chart review 1 week later revealed that fewer patients wanted CPR 36/50 (72%) and more patients 14 (28%) had an order not to have CPR (*p* = 0.007). As is typical in clinical practice, those participants who are unsure will have the default order to have CPR placed in their chart.

After the intervention, a physician discussed the CPR decision with the patient and/or family member with variable quality of patient involvement as assessed using OPTION (M = 25.66 SD 7.41, range 9–47, maximum score possible 48). These discussions were held during the patient’s dialysis treatment with others (i.e., family, nurse, pharmacist, social work, clinical coordinator, as well as other patients and their families) present in 31/49 (62%) patient conversations. Only the physician and participants were directly involved in the CPR conversation. This is usual practice as the dialysis unit is mostly an open observation unit. In the exit survey, physicians reported the CPR decision as “very relevant” to their patients (M = 3.60, SD = 0.53, range = 2–4), were “very satisfied” with the discussion they had after the intervention (M = 3.18, SD = 0.79, range = 1–4), and reported that the overall discussion was “easy” to have with their patient (M = 0.80, SD = 0.80, range = 0–3) (Table 4).

## Discussion

This was the first study to evaluate the use of the CPR-VDA specifically with patients diagnosed with ESRD. Patients with ESRD and their family members valued the CPR-VDA as a tool to help inform and consider decisions about CPR. The initial plan was to recruit patient and family dyads to participate in the study. However, it was extremely challenging to engage both partners during routine dialysis treatments. Those family members who did participate had special appointments for them to be at a specific treatment. Patients and family members found the CPR-VDA acceptable to use, even when patients’ illness and treatment caused difficulty attending to all aspects of the decision-making process all of the time. For the most part, people rated the intervention excellent, contained the right amount of information, balanced, clear, helpful, and would recommend it to others.

The CPR-VDA significantly improved knowledge about the CPR decisions. When combined with the values clarification worksheet, the CPR-VDA helped patients consider the options from their own perspective, integrate their own values, highlighted other supports and considerations, supported their confidence in decision-making, and reduced decisional conflict. Our observations are consistent with results of a recent systematic review about the effectiveness of patient decision aids [16]. Patient decision aids as a class of intervention are known to improve understanding about healthcare and treatment choices and resulting outcomes, understand risks better, integrate values with their choices, and reduce uncertainty and remorse about the decision [16].

Patients were effectively able to utilize the CPR-VDA despite lower formal educational levels. They were continuously able to attend to details in a hectic treatment environment while experiencing considerable health challenges, and varying levels of frailty. Video format patient decision aids may support patients with lower health literacy due to their oral format and flexibility since participants could start, stop, and rewind the video. Furthermore, participants were supported by both an explicit values clarification exercise and a study nurse acting as a decision coach. These findings support evidence from other studies suggesting video decision aids help patients and families make informed medical treatment decisions [25–27].

As decision coach, the study nurse provided important support to patients and families. Patient participants had difficulty navigating questionnaires due to vascular access in their arm, poor lighting, positioning for treatment, and fatigue. Thus the study nurse had a dual role in supporting them to view the video and could not help but to form a therapeutic relationship that included empathetic listening. She was able to focus on subtle changes in patient health status and adjust pacing or timing accordingly, as well as being able to give further clarification when needed. Activating other members of the healthcare team besides the physician may also be of benefit, but was not captured in this efficacy study.

Physicians reinforced the work of the study nurse and the CPR-VDA intervention during their discussion with the patient after the intervention. Thus, the study nurse as decision coach had an explicit and formal role on the interprofessional team regarding decision-making. Optimizing the decision-making environment to increase a patient’s ability to engage meaningfully in DM was a clear role for the study nurse providing decision support in this study, and could be taken on by nurses who are part of the clinical team. In this case, the study nurse was an experienced ICU nurse with additional decision coaching training. It is likely that dialysis nurses would need additional preparation or release time to take on the additional role that is fully within their scope of practice. Nurses have a clear ethical and professional obligation to support healthcare decision-making [28] and their expertise in communications and establishing therapeutic relationships could be utilized to facilitate healthcare decision-making.

While there is much to gain from understanding the use of decision-aids with patients in the dialysis setting, there are limitations to this study. The homogenous sample limits generalization of results to other populations. Recruitment of dyads of patient and family member was challenging because they were busy and not always present at dialysis appointments. Question fatigue was evident among the participants and future studies with this patient population may wish to utilize video or audio recordings to limit fatigue in answering the questions and eliminate the need of the study nurse manually recording answers during the interview. Although there was evidence of above average shared decision-making during the interview and most physicians alluded to the CPR-VDA, the assessment of the patient-physician interview was a very limited measurement as it only considered one interaction between the patient and physician. The conversation was taken out of context of any previous relationship development and / or past conversations about the subject of CPR. Measured over time and across interactions, the overall quality of shared decision-making may have been far greater. The study design would be further strengthened by adding a (randomized) control group to determine the effectiveness of the CPR-VDA intervention. In this study, the CPR-VDA was not compared to usual practice or to a control group, and did not randomize the selection of the subjects.

Preparing each member of the healthcare team to support the decision-making process may further support shared decision-making. The lack of team member participation was particularly evident by the lack of support provided to physicians by other healthcare team members. Physician members of the healthcare team working at the dialysis center had no instruction or practice using the CPR-VDA and only received a brief overview of the research project from the study nurse. Training in the use of shared decision-making and decision aids may have improved uptake and use of CPR-VDA in the clinical setting. Future interventions and pragmatic effectiveness evaluation could focus on ensuring that healthcare team members are supported to implement the CPR-VDA.

Finally, although this study was formulated around how to best support decision-making about the CPR decision, the decision-making process around life-saving interventions or end-of-life care is not just about arriving at the decision. The decision-making process involves patients and families receiving support for their grief while receiving information about loss, potential loss, or change of health status. The use of the decision aid seemed to open space for other conversations about end-of-life and grieving, which were supported by the study nurse. Anticipatory bereavement was first described by Lindemann [29] as observations of preparatory grief work done by wives with husbands at war and further conceptualized as a process to prepare terminally ill patients and their families for death thus aiding grieving [30]. However, most of the research around anticipatory bereavement involves caregivers with very little research focusing on the patients experience with end-of-life decision-making and anticipatory bereavement [31]. A future study may be able to reveal connections between the use of patient and family decision support around end-of life care and anticipatory bereavement.

## Conclusions

The CPR-VDA was feasible and acceptable to patients with ESRD, their families and the healthcare team. The CPR-VDA positively affected decision-making: improving patient and family knowledge about CPR, clarity of values, patients’ decisional self-efficacy, the congruence between documented physician’s orders and patient choice, quality of communication about CPR, while reducing decisional conflict (uncertainty) amongst patients, families, and physicians. The CPR-VDA was useful to patients regardless of their ability to engage in meaningful conversations with their family and the healthcare team about whether or not to have CPR as part of their care.

## Abbreviations

CPR: Cardiopulmonary resuscitation
CPR-VDA: Cardiopulmonary resuscitation video decision aid
ESRD: End stage renal disease
IP-SDM: Interprofessional shared decision-making model
VDA: Video decision aid
